# Infantile Epileptic Spasms Syndrome Complicating Mosaic Down‐Turner Syndrome: A Case Report

**DOI:** 10.1002/ccr3.73505

**Published:** 2026-09-09

**Authors:** Mohammad Shahrori, Khadeeja Nofal, Hasan H. Alkhawaja, Waed Mohsen, Ainaa A. Alzamari

**Affiliations:** ^1^ College of Medicine and Health Sciences Palestine Polytechnic University Hebron Palestine; ^2^ Faculty of Medicine Al‐Quds University Jerusalem Palestine; ^3^ Department of Pediatrics Ramallah Governmental Hospital Ramallah Palestine

**Keywords:** diagnostic delay, double aneuploidy, epileptic encephalopathy, infantile epileptic spasms syndrome, mosaic down‐turner syndrome

## Abstract

Severe baseline developmental delays in complex genetic syndromes like Down‐Turner mosaicism can completely mask the psychomotor regression of Infantile Epileptic Spasms Syndrome (IESS). Clinicians must maintain a high index of suspicion and prioritize early video‐EEG screening for any abnormal paroxysmal movements.

## 1 Introduction

Mosaic Down‐Turner syndrome represents an exceptionally rare double aneuploidy resulting from post‐zygotic mitotic non‐disjunction [1, 2], leading to a cellular mosaicism combining trisomy 21 (Down syndrome, DS) and monosomy X (Turner syndrome, TS) [3]. While DS is the most common autosomal abnormality globally affecting 1 in 1000 to 1100 live births [1] its concurrent manifestation with TS is estimated at a prevalence of merely 1 in 2,000,000 [3]. Superimposed on this chromosomal abnormality is Infantile Epileptic Spasms Syndrome (IESS), an age‐dependent developmental and epileptic encephalopathy characterized by the clinical triad of epileptic spasms, hypsarrhythmia, and neurodevelopmental regression [4]. Although children with DS exhibit a 10‐ to 30‐fold increased risk of developing IESS compared to the general population [5], its occurrence within a Mosaic Down‐Turner phenotype forms an exceedingly scarce clinical presentation. Phenotypically, affected patients display overlapping features of both genetic syndromes, including dysmorphic facial features and hypotonia from DS [1], alongside short stature, webbed neck, and cardiovascular anomalies characteristic of TS [2]. When complicated by IESS, the clinical presentation shifts dramatically toward frequent clusters of infantile spasms accompanied by profound loss of psychomotor milestones and loss of social engagement [4, 6]. Establishing an accurate diagnosis requires a dual neurophysiologic and cytogenetic approach. High‐resolution karyotyping and fluorescence in situ hybridization (FISH) are essential to quantify the distinct cell lines [3], while prolonged video‐electroencephalography (video‐EEG) is required to identify pathognomonic hypsarrhythmia [4]. Therapeutic management demands prompt intervention to halt spasms and preserve cognitive function. First‐line therapies include adrenocorticotropic hormone (ACTH) and vigabatrin to which trisomy 21 patients typically demonstrate high responsiveness [5] integrated with multidisciplinary cardiac, endocrinological, and developmental surveillance [1, 2, 6]. This report highlights a rare case of IESS in a patient with Mosaic Down‐Turner syndrome, aiming to discuss the clinical diagnostic complexities, the challenges of masking phenotypes, and therapeutic strategies.

## 2 Case History/Examination

We present the case of a 2‐year and 4‐month‐old female toddler who was admitted to the Pediatric Department of Palestine Medical Complex Hospital for the evaluation and management of recurrent epileptic spasms. The patient was born at 37 weeks of gestation via normal vaginal delivery without peri‐ or post‐natal complications. Her birth weight was 1900 g, and she did not require neonatal intensive care. The patient has non‐consanguineous parents and six healthy siblings. There is no history of epilepsy, developmental disorder, genetic syndromes, and congenital disorders among her family members. Her past medical history was significant for recurrent vomiting since birth, attributed to a suspected cow's milk protein allergy, for which she was maintained on an amino acid‐based formula. She was additionally diagnosed with subclinical hypothyroidism. An echocardiogram revealed congenital heart defects, including left ventricular hypertrophy and a small patent foramen ovale (PFO). Developmentally, the patient exhibited severe global developmental delay; she was unable to achieve age‐appropriate milestones such as independent sitting and meaningful speech, although she remained socially active and tolerated pureed food. At the age of 6 months, the patient developed abnormal stereotypic motor episodes characterized by sudden episodes of increased tone involving the upper and lower extremities. The episodes occurred mainly during sleep and consisted of bilateral extension of the upper and lower limbs occurring in clusters, with each cluster lasting approximately 5 min. Initially, these episodes occurred once daily and were not associated with a postictal state. Over the 3 months prior to the current admission, the frequency and nature of the episodes had changed, from once daily to approximately 3 times per day. The events were more characteristic for epileptic spasms, consisting of sudden forward flexion of the trunk with extension of both upper and lower extremities occurring in clusters lasting about 5 min. On physical examination, the patient was alert, active, well‐hydrated, and in no acute distress with stable vital signs. Facial dysmorphic features typical of Down syndrome included epicanthal folds, upslanting palpebral fissures, midfacial hypoplasia, small low‐set ears, and macroglossia. Neurologic examination showed marked axial hypotonia with relatively preserved peripheral muscle tone. The remainder of the physical examination was unremarkable.

## 3 Differential Diagnosis, Investigations and Treatment

**FIGURE 1:**
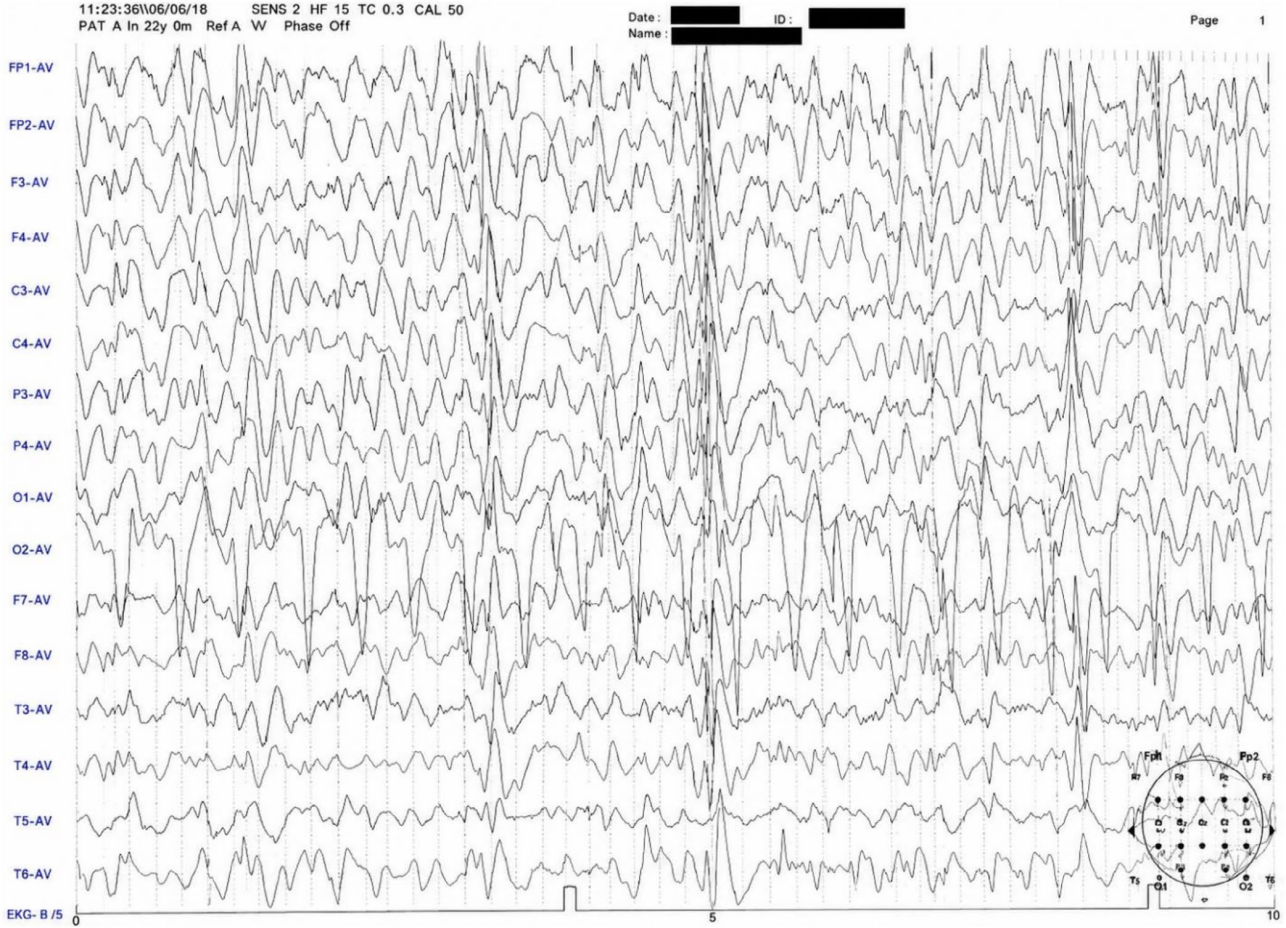
Prolonged video‐EEG demonstrating classic hypsarrhythmia and ictal features of Infantile Epileptic Spasms Syndrome (IESS).

During the initial evaluation of these developmental and neurological abnormalities, peripheral blood karyotyping was performed and revealed mosaic Down–Turner syndrome with the following karyotype (47, XX, +21[17]/45, X [23]). The patient was initially started on oral levetiracetam at a dose of 70 mg twice daily for 1 month; however, the medication was discontinued by the family against medical advice. The abnormal movements persisted. Approximately 1 year and 5 months after symptom onset, a prolonged EEG was successfully performed and demonstrated classic hypsarrhythmia, confirming the diagnosis of Infantile Epileptic Spasms Syndrome (IESS; Figure 1).

Brain imaging (CT or MRI) was not performed due to limited resources and the primary reliance on EEG and genetic confirmation. During hospitalization, the epileptic spasms were directly observed and documented by the pediatric neurology team. The patient received an intravenous loading dose of levetiracetam (400 mg IV). Subsequently, a high‐dose oral prednisolone regimen (15 mg/5 mL formulation) was initiated at 10.5 mg every 6 h for 2 weeks. This was followed by a structured tapering protocol: 10.5 mg twice daily for 5 days, 10.5 mg once daily for 5 days, and 4.5 mg once daily for 3 days prior to complete discontinuation.

## 4 Discussion

Infantile Epileptic Spasms Syndrome (IESS), formerly known as West syndrome and recently reclassified by the International League Against Epilepsy (ILAE), was first described by Dr. William James West in 1841 when he observed the condition in his own son [7]. While specific Middle Eastern epidemiological data are scarce, the region exhibits a high prevalence of pediatric epileptic encephalopathies [8]. Children with Trisomy 21 carry a 10‐ to 30‐fold increased risk of developing IESS compared to the general population [5]. IESS is etiologically classified into three categories: structural, metabolic, and genetic (formerly symptomatic), and unknown etiology (formerly cryptogenic) [4]. Known risk factors include hypoxic–ischemic encephalopathy (HIE), central nervous system infections, structural brain anomalies, and chromosomal aberrations, most notably Trisomy 21 [6]. Pathogenetically, the extra chromosome 21 leads to overexpression of specific genes, causing impaired migration of GABAergic interneurons and altered synaptogenesis [5, 9]. Conversely, Turner syndrome lacks a distinct association with infantile spasms [2]. The exact pathogenesis of IESS remains partially understood, but two major hypotheses predominate. The first is the corticotropin‐releasing hormone (CRH) excess theory, which suggests that stress‐induced dysfunction in the brainstem leads to an overproduction of CRH, triggering spasms [10]. In our patient, the Trisomy 21 cell line likely drove the epileptogenic process, while the Monosomy X (Turner) cell line contributed primarily to the cardiovascular anomalies (PFO, LVH) and early growth restriction, as Turner syndrome is not classically associated with epileptic encephalopathies [2]. The classic clinical manifestation of IESS involves the triad of epileptic spasms, psychomotor regression, and hypsarrhythmia on EEG. Spasms typically emerge between 3 and 12 months of age and can be flexor, extensor, or mixed, typically occurring in clusters upon awakening or during sleep transitions [4, 6]. In our case, the onset at 6 months aligns with typical presentation. However, the cardinal sign of “regression” was heavily masked by the profound baseline global developmental delay inherent to the Down‐Turner phenotype. This masking effect can easily lead to misdiagnosis, necessitating careful differentiation from other paroxysmal events in infancy. The definitive diagnosis of IESS relies heavily on clinical observation of the spasms coupled with neurophysiological confirmation. A prolonged video‐EEG is the gold standard, characteristically revealing hypsarrhythmia, a chaotic, high‐voltage pattern of slow waves intermixed with multifocal spikes [4]. In this case, while a brain MRI was omitted due to resource limitations, the confirmation of the genetic etiology via karyotyping (47, XX, +21[17]/45, X [23]) alongside a positive EEG provided a highly definitive diagnostic framework, justifying the initiation of therapy without further neuroimaging delays. Therapeutic protocols for IESS prioritize the rapid cessation of spasms and resolution of hypsarrhythmia to mitigate further cognitive deterioration. First‐line therapies include hormonal treatments (ACTH or high‐dose oral corticosteroids) and vigabatrin. Vigabatrin is particularly favored for patients with tuberous sclerosis complex, whereas hormonal therapies are often preferred for other etiologies, including Trisomy 21 [6]. Interestingly, literature suggests that children with Down syndrome and IESS respond more favorably to first‐line therapies compared to those with other structural etiologies [5]. Despite a significant diagnostic delay of over 1.5 years, our patient demonstrated a remarkable and rapid cessation of clinical spasms upon the initiation of a structured high‐dose oral prednisolone tapering regimen. The initial brief and likely underdosed trial of levetiracetam was ineffective, which is consistent with guidelines that do not recommend levetiracetam as a first‐line monotherapy for infantile spasms [4]. The intersection of these three conditions is statistically extraordinary. Mosaic Down‐Turner syndrome has an estimated prevalence of merely 1 in 2,000,000 [3]. Superimposing IESS onto this already exceedingly rare genetic mosaicism forms an almost unprecedented clinical triad. To the best of our knowledge, this is the first reported case in the literature of mosaic Down‐Turner syndrome with associated Infantile Epileptic Spasms Syndrome. This case underscores that severe underlying genetic syndromes can obscure the onset of epileptic encephalopathies, highlighting the critical need for early EEG screening when evaluating abnormal movements in infants with delayed milestones.

## 5 Conclusion and Results (Outcome and Follow‐Up)

The patient demonstrated significant clinical improvement with complete cessation of the spasms following the initiation of corticosteroid therapy and was discharged home in stable condition with a structured outpatient follow‐up plan. The concurrence of IESS within a Mosaic Down‐Turner phenotype represents an extraordinarily rare clinical intersection. This case underscores the significant diagnostic challenges encountered when epileptic encephalopathies arise in patients with complex genetic syndromes. Profound baseline neurodevelopmental delays, inherent to double aneuploidies like Down‐Turner mosaicism, can completely mask the classic psychomotor regression that typically heralds IESS. Consequently, pediatricians and neurologists must maintain a high index of suspicion and prioritize early prolonged video‐EEG screening when evaluating any abnormal, repetitive, or stereotypic paroxysmal movements in this vulnerable population. Furthermore, this report reaffirms that despite substantial diagnostic delays, patients with Trisomy 21‐driven epileptogenesis can still demonstrate an excellent and rapid therapeutic response to first‐line high‐dose corticosteroid protocols.

## Author Contributions

**Mohammad Shahrori:** conceptualization, investigation, data curation, writing – original draft, project administration. **Khadeeja Nofal:** investigation, data curation, writing – original draft. **Hasan H. Alkhawaja:** validation, visualization, writing – review and editing. **Waed Mohsen:** supervision, conceptualization, validation, writing – review and editing. **Ainaa A. Alzamari:** validation, writing – review and editing.

## Funding

The authors have nothing to report.

## Consent

Written informed consent was obtained from the patient's parents for the publication of this clinical case report and any accompanying non‐identifying clinical details.

## Data Availability

Data sharing not applicable to this article as no datasets were generated or analysed during the current study.
